# Novel Surfactant-Free Water Dispersion Technique of TiO_2_ NPs Using Focused Ultrasound System

**DOI:** 10.3390/nano11020427

**Published:** 2021-02-08

**Authors:** Seon Ae Hwangbo, Minjeong Kwak, Jaeseok Kim, Tae Geol Lee

**Affiliations:** Nanosafety Team, Safety Measurement Institute, Korea Research Institute of Standards and Science (KRISS), 267 Gajeong-ro, Yuseong-gu, Daejeon 34113, Korea; hbsa@kriss.re.kr (S.A.H.); kwakmj@kriss.re.kr (M.K.); jaeseok.kim@kriss.re.kr (J.K.)

**Keywords:** TiO_2_ colloid, ultrasonic dispersion, particle size distribution, colloid stability, pH, zeta potential

## Abstract

Titanium dioxide (TiO_2_) nanoparticles are used in a wide variety of products, such as renewable energy resources, cosmetics, foods, packaging materials, and inks. However, large quantities of surfactants are used to prepare waterborne TiO_2_ nanoparticles with long-term dispersion stability, and very few studies have investigated the development of pure water dispersion technology without the use of surfactants and synthetic auxiliaries. This study investigated the use of focused ultrasound to prepare surfactant-free waterborne TiO_2_ nanoparticles to determine the optimal conditions for dispersion of TiO_2_ nanoparticles in water. Under 395–400 kHz and 100–105 W conditions, 1 wt% TiO_2_ colloids were prepared. Even in the absence of a surfactant, in the water dispersion state, the nanoparticles were dispersed with a particle size distribution of ≤100 nm and did not re-agglomerate for up to 30 days, demonstrating their excellent dispersion stability.

## 1. Introduction

Along with SiO_2_ and ZnO nanoparticles, TiO_2_ nanoparticles are one of the most abundantly produced nanomaterials. Annually, approximately 3 million tons of TiO_2_ are produced industrially across the world for use in photocatalysts, self-cleaning agents, UV-protection agents, self-cleaning ceramics, antibacterial air purifiers, water treatment catalysts, cosmetics, inks, and packing materials [1,2]. Because of their wide range of applicability, controlling particle dispersion and agglomeration in the colloids is crucial for determining the properties and characteristics of the TiO_2_ nanoparticles for controlling product performance. Compared with microparticles, nanoparticles exhibit a stronger attraction between particles (van der Waals force); thus, they possess enough energy to form agglomerates [3]. Due to this issue, the size and surface treatment of each particle is different, depending on the relevant application field; however, ideally, the physicochemical properties of the surface should remain unaltered. A typical example is that of sunscreen lotion. Based on the current production process, the particles in sunscreen must be uniformly dispersed at a size <100 nm and in a non-re-agglomerated colloidal state to maintain stability. However, to prepare such suspensions, surfactants or dispersing agents are often used, which alter the physical and chemical properties of TiO_2_ surfaces; this necessitates the addition of viscosity controlling agents, which are detrimental to the environment and human body. The addition of these agents also results in additional production costs.

Conventional physical and mechanical dispersion methods, such as ball and jet milling, contaminate the dispersion because of a high milling speed and are ineffective in achieving complete dispersion of the nanoparticles [4,5]. As a result, contactless dispersion methods using ultrasonic dispersion equipment are frequently employed. Ultrasonic dispersion is an effective tool for controlling aggregation, and considerable research has been conducted on the irradiation and measurement conditions of ultrasonic waves [6,7]. However, despite the growing interest in ultrasonic dispersion, there is lack of sufficient systematic research regarding these methods [3].

The most common ultrasound dispersion methods bath, horn, and cup dispersions are not conducive to nanoscale applications because of their low energy, non-uniform dispersions, and uncontrollable heating during operation [8,9,10], necessitating the use of surfactants for commercial products due to their significantly low dispersion stability [11,12]. Since TiO_2_ is a relatively cost-efficient material used in a wide variety of applications, dispersion methods that can maintain the stability of TiO_2_ colloids play an important role in increasing its potential for use in large industrial applications. Therefore, it is essential to investigate the influence of ultrasonic radiation on the dispersion and stability of TiO_2_ colloidal nanoparticles [13,14]. This study focuses on the effects of ultrasonic irradiation on the dispersion properties of TiO_2_ colloids by evaluating the dispersion stability and further suggests optimal TiO_2_ dispersion protocols.

This study investigated a novel surfactant-free water dispersion technique on TiO_2_ nanoparticles using focused ultrasound and proposed an optimum TiO_2_ dispersion protocol. By using the proposed protocol to prepare waterborne TiO_2_ nanoparticles, this study prepared waterborne TiO_2_ nanoparticles with a particle size distribution of ≤100 nm that resisted re-agglomeration for up to 30 days, demonstrating their excellent dispersion stability.

## 2. Materials and Methods

### 2.1. Materials

Details on the TiO_2_ nanoparticles, solvents, and TiO_2_ colloids used in the experiment are provided in Table 1.

### 2.2. Dispersion Method

**Figure 1:**
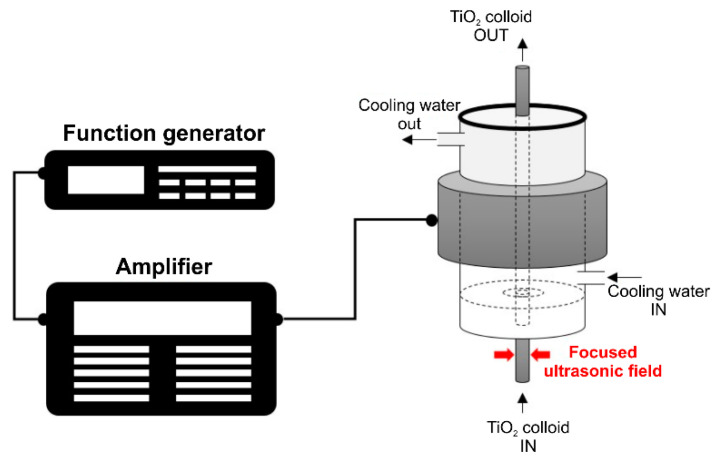
Schematic of the ultrasonic dispersion equipment employed in this study.

This study used a focused ultrasonic dispersion method for the water dispersion of the surfactant-free TiO_2_ nanoparticles [15,16]. In this method, acoustic energy is focused onto the center of a cylindrical piezoelectric ceramic. Unlike the existing bath or horn ultrasound methods, an extremely high level of energy is focused onto the center because a high frequency of approximately 400 kHz is used. Furthermore, as the driving temperature of the equipment is controlled through cooling water, long-time driving is feasible to achieve the nanoparticle dispersion up to the desired scale. Figure 1 shows a schematic diagram of the ultrasonic dispersion equipment employed in this study.

**Figure 2:**
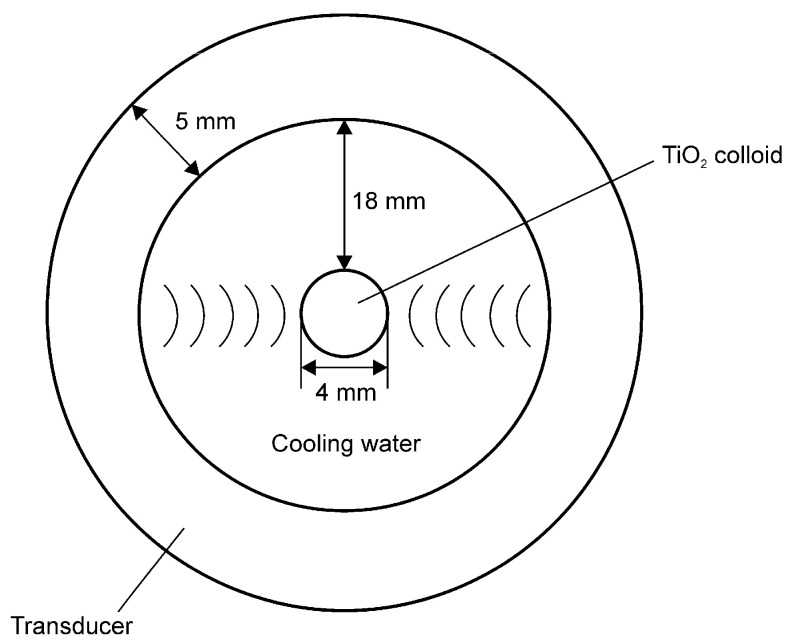
Aerial cross-sectional view of the ultrasonic dispersion equipment and the dimensions of the focused ultrasonic field.

The amplified energy is transferred to the piezoelectric ceramic through a function generator and amplifier, and the sound energy is concentrated in the center of the cylinder. Dispersion occurs by passing the suspension through the focused ultrasonic wave, and because all the fluid circulates and passes through this section, uniform particle dispersion is rendered possible. In addition to controlling the heat generated during sonication, the cooling water circulating through the piezoelectric ceramic (PZT) acts as a medium to generate the ultrasonic waves and deliver the sound pressure to the center of the colloid sample; this provides a way to control the dispersion conditions. The detailed dimensions of the focused ultrasonic field are shown in Figure 2. Adjusting the thickness and size of the cylindrical transducer allows changing the desired frequency. A resonance frequency of 396 kHz was used in the experiment.

### 2.3. Acoustic Pressure and Streaming

**Figure 3:**
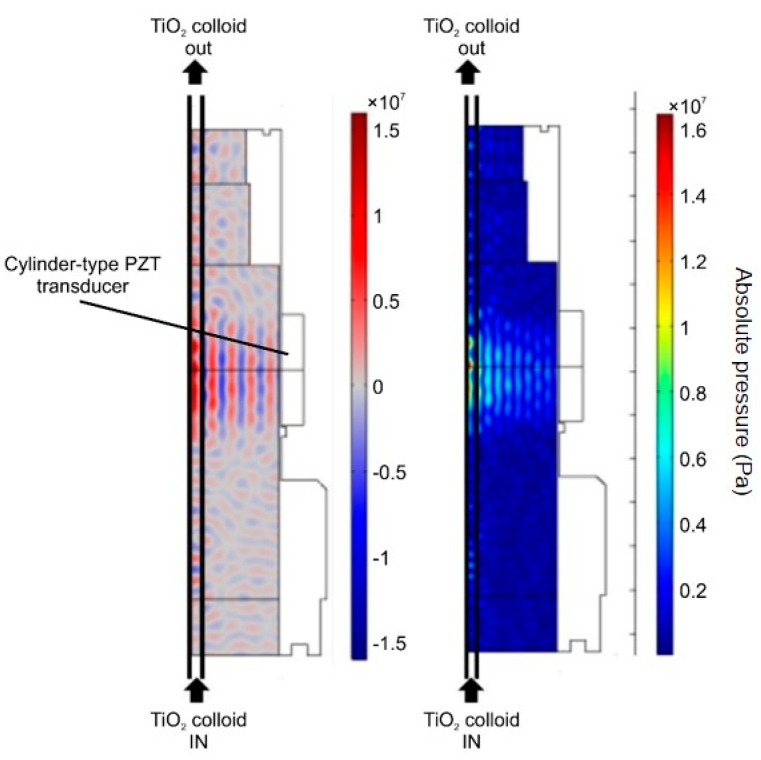
Sound pressure (**left**) and energy (**right**) distribution models of the focused ultrasound radiation.

Acoustic streaming of the proposed system was simulated using the COMSOL Multiphysics software (version 4.2, COMSOL Multiphysics®, Stockholm, Sweden) [17]. The acoustic pressure gradient and the actual energy distribution pattern of the ultrasonic dispersion equipment used in this study (Figure 3) clearly depict the focused sound pressure and the sound energy distribution.

**Figure 4:**
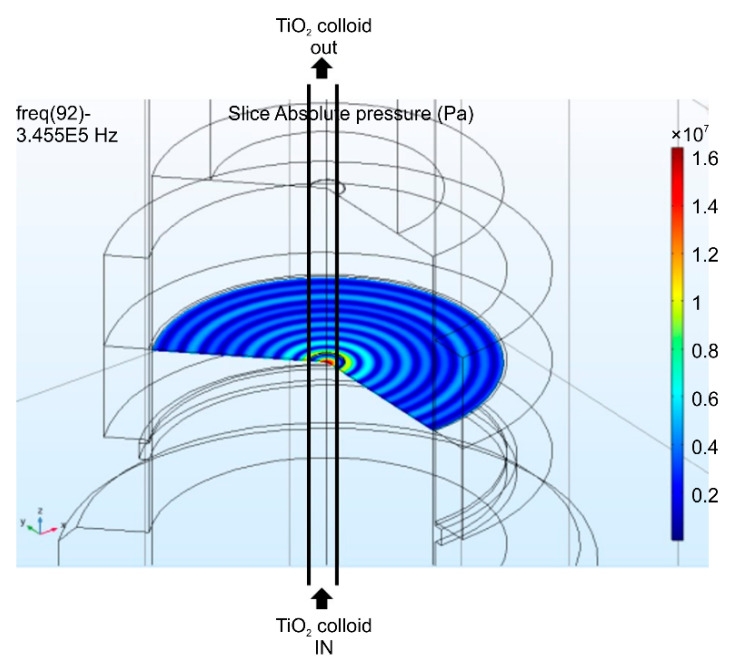
Three-dimensional sound field modeling of the focused ultrasound radiation.

Figure 3 shows the cross-sectional view of the focused instrument. Dispersion is induced by passing the sample through the black line on the left. Both pressure and energy are concentrated at the cylindrical center, and both sound pressure and energy are highest at the left line, through which the sample fluid passes. Figure 4 shows a three-dimensional shape simulation, which indicates that the energy is highly concentrated in the cylindrical center. We expect this method to be more efficient for nanoparticle dispersion.

### 2.4. Characterization of TiO_2_ Colloid

#### 2.4.1. SEM and TEM

Scanning electron microscopy (SEM; Zeiss GeminiSEM 500, Jena, Germany) and transmission electron microscopy (TEM; JEM-ARM200F, JEOL, Freising, Germany) were performed to determine the changes in the aggregated state before and after the ultrasonic dispersion of the TiO_2_ nanoparticle colloid. The overall aggregation distributions before and after ultrasonic dispersion were investigated through SEM images at low magnification, and the distribution state of the nanoparticles was determined through the TEM images at high magnification.

For SEM analysis, the suspension (100 μL) was diluted by a factor of 10 with deionized water and added dropwise onto a Piranha-cleaned silicon wafer. SEM images were captured at an acceleration voltage of 5~10 kV and an operating distance of 3.7 mm.

For TEM analysis, a sample was prepared by diluting a suspension (100 μL) by a factor of 10 with deionized water and further dehumidifying it for 24 h in a desiccator at 25 °C after loading the sample onto a Lacey Carbon film (300 mesh copper). TEM images were captured at an acceleration voltage of 150 kV.

#### 2.4.2. Size Distribution, pH, and Zeta Potential

Particle size distribution, pH, and zeta potential were measured to observe changes in the dispersion sample over various periods of ultrasound exposure. Particle size distribution was analyzed using a centrifuge particle sizer (CPS, DC 24,000) using centrifugation because, compared with the conventional laser diffraction method, this approach more accurately analyzes the particle sizes of the dispersed samples [18]. The size distribution of the particles in the colloid depends on both the particle size and the weight and was measured at different centrifugal forces. For particle size measurement, 1 wt% of the dispersion sample was diluted 20-fold with deionized water, the rotational speed of the particle size analyzer (CPS) was analyzed at 24,000 rpm. To maintain stability of the dispersed particles, it is important that they be kept apart from the electrostatic forces. Thus, the changes in colloid stability were determined by measuring the pH (HORIBA, LAQUA F-71) and zeta potential (Malvern, Zetasizer Nano ZS, Malvern Panalytical, Malvern, UK), as they are the representative indicators of electrostatic stabilization of colloids [19].

## 3. Theory/Calculation

### 3.1. Particle Mobility in a Colloid

Particles in an aqueous solution exhibit several behaviors, which can be classified into gravitational behavior and particle molecular motion, i.e., Brownian motion. Particles with a specific gravity greater than that of water move from the top to the bottom of a suspension over time via a process referred to as precipitation. In addition, particles in an aqueous solution have their own molecular velocity that must be considered when describing their physical properties and the viscosity of the aqueous solution. The sedimentation rate due to gravity (Equation (1)) and the velocity of Brownian motion (Equation (2)) are described below [20].

(1) $$V=\frac{2gr^{2}↩p}{9\eta }$$

(2) $$\Delta =\left(\frac{kTt}{3\pi \eta r}\right)$$

Here, V is the distance moved by the particle in *t* seconds, *g* is the gravity, *r* is the particle radius, ↩p is the density difference between the dispersed phase and dispersion medium, *η* is the viscosity of the dispersion medium (continuous phase), Δ is the distance at which particles start moving in *t* seconds, *k* is the Boltzmann integer, and *T* is the absolute temperature of the dispersion medium (solvent).

Equations (1) and (2) show that the behavior of the particles in an aqueous solution is affected by particle size. The sedimentation rate of particles with a higher specific gravity than that of water is proportional to the square of the particle radius. On the other hand, the Brownian motion velocity is inversely proportional to the particle radius. It can be predicted that particles with higher specific gravity than that of water will exhibit faster sedimentation and slower Brownian motion with increasing particle size in an aqueous solution, whereas smaller particles will exhibit slower sedimentation and faster Brownian motion. By controlling the particle size, the behavior of the particles as well as dispersion stability in an aqueous solution can be controlled. Therefore, controlling particle size is a key factor in producing a stable colloid. As previously discussed, although particle size can be controlled using surfactants or other chemical dispersants, they can also alter the particle properties. Thus, dispersing particles in a suspension without using dispersants would be a key breakthrough.

### 3.2. Mechanisms of Ultrasonic Dispersion

**Figure 5:**
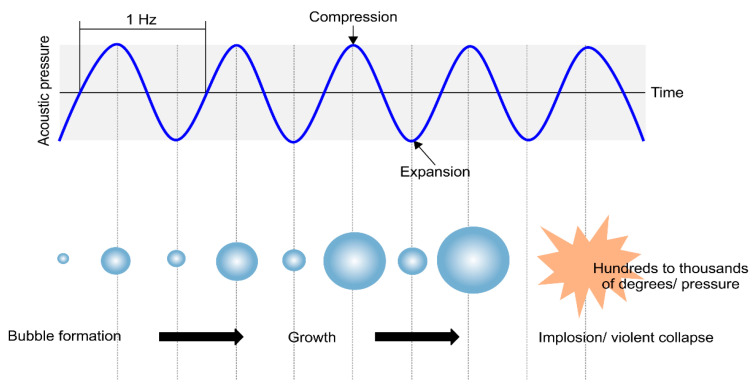
Process of cavitation generation and collapse via ultrasound.

Cavitation, which is used in ultrasonic dispersion, is generated by the application of a repeated amount of sound pressure per second. An ultrasonic wave is generated, and cavitation bubbles become enlarged while the sound pressure is repeatedly applied. When the bubbles grow to the critical value, they collapse. The temperature and pressure of the generated bubbles can vary from hundreds to thousands of degrees Kelvin and atmospheres, respectively. The temperature and atmospheric pressure generated during bubble collapse are referred to as the collapse power, and their values vary depending on the given frequency, ultrasonic irradiation time, temperature, medium type, and voltage. Figure 5 shows a schematic of the process of bubble generation and collapse during ultrasonic irradiation in a liquid medium. Ultrasonic waves irradiated through the liquid medium produce compressive and tensile stresses that cause the microbubbles to shrink and expand, respectively.

The energy generated by collapsing bubbles can deliver a constant or varying energy to its surrounding medium [21,22], depending on the frequency, temperature, power, and viscosity of the medium. This energy can be used to clean surfaces, disperse agglomerated particles, and emulsify water and oil. It is crucial to ascertain the conditions for ultrasonic dispersion because the temperature and pressure generated during collapse vary according to different conditions.

### 3.3. Calculations

#### 3.3.1. Calibration of Ultrasonic Energy by the Calorimetric Method

Ultrasonic energy may be calculated using the calorimetric method, which determines the energy based on temperature changes in the solvent. The energy generated through the ultrasonic wave, or more precisely, the power of the ultrasonic wave generated per second, can be calculated by using Equation (3) [23,24].

(3) $$P=\frac{dT}{dt}MC_{p}.$$

Here, *T* is the solution temperature (K), *C_p_* is the heat capacity of water (4.186 J/g °K), and *M* is the mass of the solution (g = mL). The *dT/dt* values were calculated using twenty temperature plots during the 30-min-long temperature convergence. The input frequency (f) and power (Pset) used for dispersion were 396 kHz and 102 W, respectively, while the ultrasonic energy (Pult) was calculated to be 27.048 W (J/s).

#### 3.3.2. Deliveries of Sonic and Critical Sonic Energies

It is important to ensure that small particles are uniformly present in the colloid and that no re-agglomeration occurs during dispersion. The critical ultrasonic energy required to obtain the most stable colloid with the most uniform and narrow particle size distribution depends on the properties of the material and the colloid. Delivery of sonic energy (DSE, J/mL) is the final ultrasonic energy imparted to the dispersion medium, which may be calculated using Equation (4) according to the ultrasonic irradiation time [25].

(4) $$DSE=P\times \frac{t}{V}.$$

Here, *DSE* is the delivery of s.nic energy (J/mL), *P* is the ultrasonic energy determined by calorimetric method (J/s), *t* is the irradiation time (sec), and *V* is the solution volume (g = mL).

The DSE value can be obtained according to the ultrasonic irradiation time. When the colloid maintains a stable state without re-agglomeration at a specific DSE value, this value becomes the delivery of the critical sonic energy (DSEcr, J/mL) of the colloid [25]. DSEcr (critical delivered sonication energy) refers to the optimum energy to maintain dispersion stability without damaging the material. In the ultrasonic dispersion experiment, research on selection of the optimal DSEcr value through analysis of dispersion stability and properties of the material after dispersion for various ultrasonic irradiation energies should always be conducted together. Research in selecting the optimal DSEcr value via dispersion stability analysis and properties of the material after dispersion for various ultrasonic irradiation energies should always be conducted together. If energy above this value is applied to the colloid, the material may be damaged. Therefore, it is crucial to determine the appropriate DSEcr for the material in order to obtain a stable dispersion without deforming the material [25].

## 4. Results and Discussion

### 4.1. SEM and TEM

**Figure 6:**
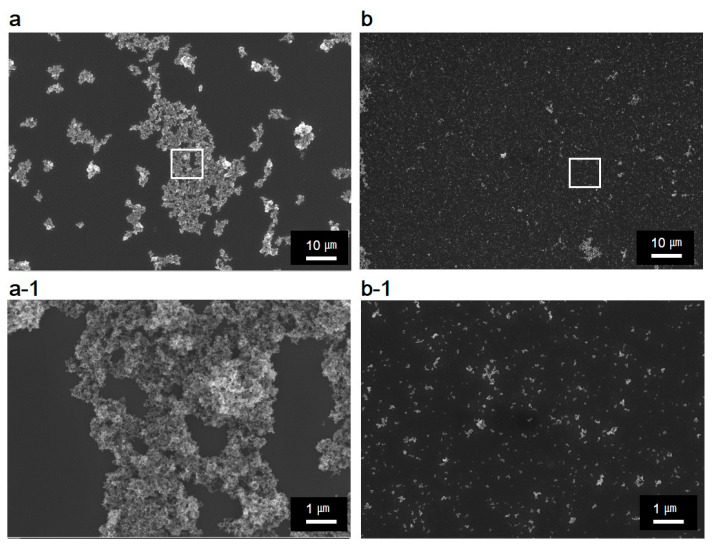
SEM images before (**a**) and after (**b**) ultrasonic dispersion. (**a-1**) and (**b-1**) represent the magnified images of the regions marked in white boxes in (**a**) and (**b**), respectively.

Figure 6 shows the SEM images of the TiO_2_ colloidal nanoparticles before and after ultrasonic dispersion; Figure 6a,b show 1000-times magnified images of the TiO_2_ nanoparticles recorded at an acceleration voltage of 10 kV. The region marked with a white box was further observed at a 10,000-times magnification and an acceleration voltage of 5 kV (Figure 6(a-1,b-1)). As shown in the magnified images, the samples predominantly contained agglomerations before the ultrasonic dispersion. On the other hand, the TiO_2_ nanoparticles were well-distributed after ultrasonic dispersion, and the corresponding magnified image (Figure 6(b-1)) shows a less agglomerated distribution than that observed before the ultrasonic dispersion.

**Figure 7:**
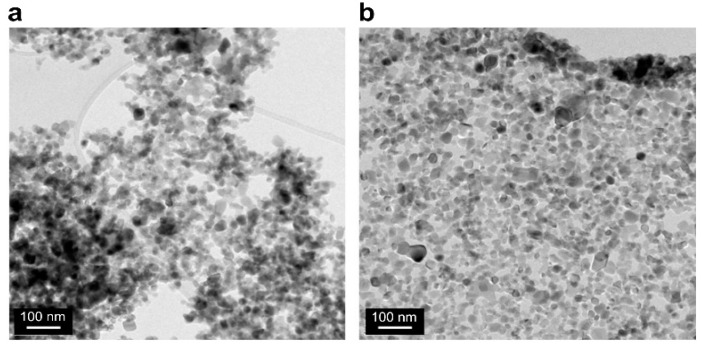
TEM images before (**a**) and after (**b**) ultrasonic dispersion.

The SEM images (Figure 6) clearly reveal that TiO_2_ nanoparticles were more evenly distributed after ultrasonic dispersion than those before the dispersion. Figure 7 shows the TEM images of the TiO_2_ nanoparticles before and after ultrasonic dispersion. While the SEM images show the overall agglomeration changes before and after dispersion, the TEM images allow a more detailed observation of particle agglomeration with magnifications at the 100 nm scale. As Figure 7a suggests, most of the nanoparticles agglomerated as colloids by van der Waals forces. However, the agglomeration was diminished significantly after ultrasonic dispersion, as shown in Figure 7b, where the particles seldom overlap. This was due to the focused, high-intensity ultrasound waves, which deagglomerated the nanoparticles and induced an even distribution.

As shown in Figure 6 and Figure 7, the focused ultrasound technique is a powerful method that can disassemble nanoparticle agglomeration. Thus, ultrasonic dispersed nanoparticles were also analyzed using XRD not only because the particles could become separated, but also because a change in their crystal structures was a concern. It was observed that the crystal structures of the TiO_2_ nanoparticles did not alter after ultrasonic dispersion and that the ratio between anatase and rutile also remained unchanged. XRD results are provided in Figure S1 (Supplementary Materials).

### 4.2. Particle Size Distribution

**Figure 8:**
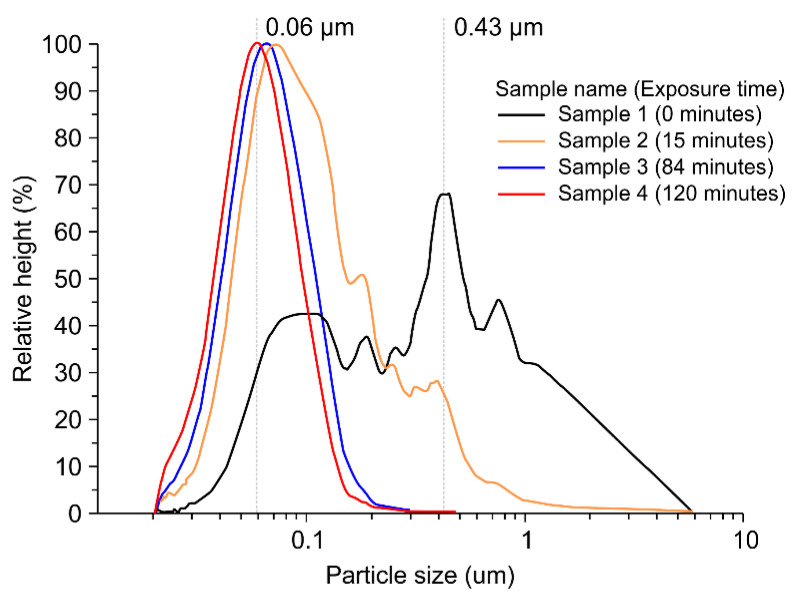
Particle size and size distributions of TiO_2_ particles for different ultrasonic exposure times (samples 1 (black, 0 min), 2 (yellow, 15 min), 3 (blue, 84 min), and 4 (red, 120 min)).

The degree of colloidal dispersion was determined by particle size distribution measurements, according to the duration of ultrasound exposure. Figure 8 shows the size distributions of TiO_2_ nanoparticles measured before and after ultrasonic dispersion.

The ultrasonic wave exposure time increased in the following order: 0 min (black line), 15 min (yellow line), 84 min (blue line), and 120 min (red line). The range of the particle size distribution became narrower, and the modal value decreased with increasing exposure time to ultrasonic waves. The particle sizes before ultrasonic dispersion (black line) were broadly distributed in a coagulated state, with particles 0.03−6 μm in diameter. In particular, the particles existed in a variety of sizes in the colloid, and the agglomerate sizes were distributed over several microns, which was unsuitable for a process requiring a uniform dispersion. Because of their distribution, the attraction between the particles was so high that they were extremely unstable and possessed a high probability of re-agglomeration or precipitation. With increasing exposure time to ultrasonic waves, the particle size distribution became narrow, and the particle size decreased. The sample with complete dispersion (sample 4) exhibited an extremely narrow and highly uniform particle size distribution compared with the other samples, and its modal value of 0.06 μm exhibited a single peak. The distribution of several small particles can be maintained for an extended amount of time without being precipitated by increasing the speed of Brownian motion beyond the settling velocity of particles in the colloid; this implies that the probability of re-agglomeration of sample 4 is extremely small compared with that of sample 1 as it can be maintained in a highly stable state for a long time.

Table 2 shows the DSE, average particle size, and polydispersity index (PDI) of the TiO_2_ colloids according to the focused ultrasonic exposure times.

The changes in the DSE values and particle sizes were determined based on the samples collected from TiO_2_ colloids (1 wt%) at six different ultrasound exposure times ranging from 15 min to 120 min. The average particle size before ultrasonic dispersion was 0.783 µm, and the PDI value was 24.263, indicating the formation of colloids with a broad particle size distribution. However, as the ultrasound exposure time increased, the average particle size decreased. As shown in Table 2, re-aggregation occurred over time after the DSE of the irradiated ultrasonic dispersion in the colloids reached up to 1753 J/mL, while the colloids irradiated with a *DSE* of 1947 J/mL maintained stability without any change in the average particle size. This result suggests that the DSEcr, which is the energy required for manufacturing stable waterborne TiO_2_ (1 wt%) nanoparticles, is 1947 J/mL. Furthermore, as the ultrasonic dispersion time increased, the particle size and size distribution, as well as the PDI, decreased, indicating that the nanoparticles were distributed as TiO_2_ colloids with better dispersity and narrow particle size distribution. Thus, by using these optimal conditions (120 min of exposure with a DSEcr = 1947 J/mL, f = 396 kHz, and Pset = 102 W) for the ultrasonic dispersion method, stable TiO_2_ (1 wt%) colloids (an average particle size of 0.068 μm and PDI of 1.51) were obtained.

### 4.3. Zeta Potential and pH

**Figure 9:**
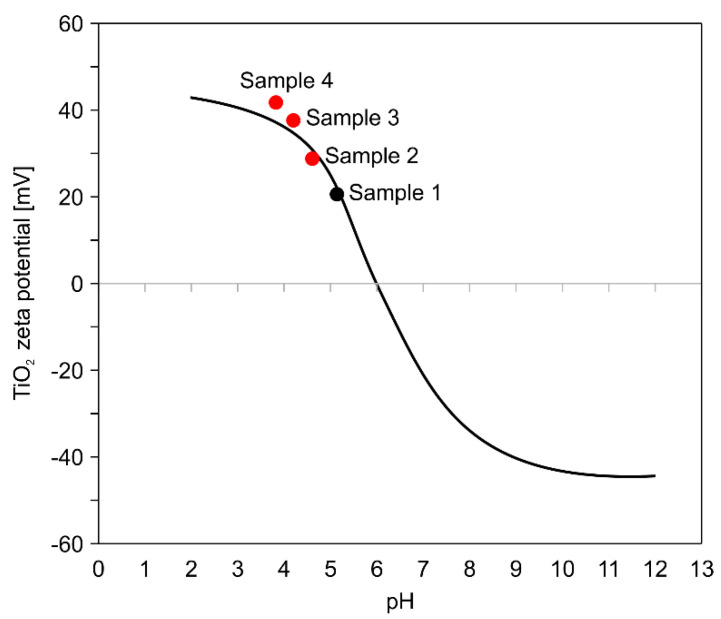
Changes in pH and zeta potentials of TiO_2_ colloid samples such as sample 1 (0 min), sample 2 (15 min), sample 3 (84 min), and sample 4 (120 min) after various ultrasonic wave exposure times.

The zeta potential is a major indicator of colloidal dispersion stability. The magnitude of the zeta potential indicates the degree of electrostatic repulsion between similarly charged adjacent particles in the dispersed system. A high zeta potential will stabilize small molecules and particles; thus, a colloid with a high zeta potential can resist agglomeration. If the zeta potential is small, the attractive force can exceed the repulsive force, thereby breaking the suspension and leading to agglomeration. When the zeta potential is less than ~35 mV, cohesion or stability is generally broken, and when the potential is between ±40 and ±60 mV, the stability is evaluated to be excellent [26,27]. The curve in Figure 9 shows the relationship between the pH and zeta potential of the TiO_2_ colloid [28]. The four points represent the pH and zeta potentials of the colloid before and after dispersion.

Sample 1 represents the zeta potential value before ultrasonic dispersion and samples 2–4 represent the zeta potential values after exposure to ultrasound for 15 min, 84 min, and 120 min, respectively. For sample 4, the zeta potential is expected to be ~40 mV at pH 3.8; however, it lies above the curve (43.1 mV) because of its more stable state due to ultrasonic dispersion. Ultrasonic dispersion ensures that particles are dispersed into particles of sufficiently small size in the colloid and that the double-layer formation between the solvent and particles creates a repulsive force between the particles, thus maintaining the colloids in a stable state of without prolonged re-agglomeration.

As shown in Table 3, the zeta potential increases from 19.6 to 43.1 mV with increasing DSE. The TiO_2_ colloid can maintain its stable state after ultrasonic dispersion, and colloids exposed to a DSE of 1947 J/mL exhibit excellent dispersion. Detailed information for each colloid can be found in Table 3.

### 4.4. Stability of TiO_2_ Colloids

**Figure 10:**
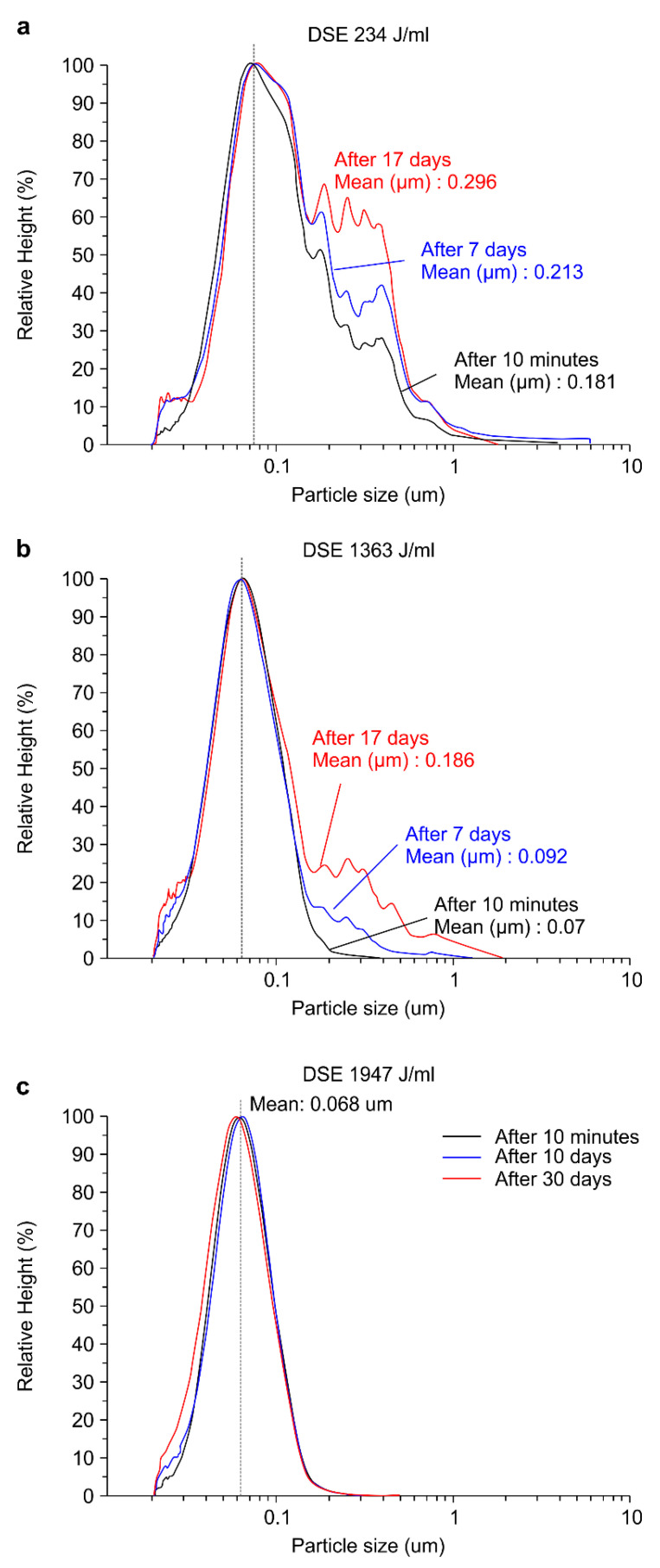
Particle size, size distributions, and stability of TiO_2_ colloids with DSE values of (**a**) 234, (**b**) 1363, and (**c**) 1947 J/mL.

The purpose of dispersion is to obtain an extremely small and uniform hydrodynamic diameter of the particles in the colloid, while increasing the duration of its stability by preventing changes and re-agglomeration. Employing the optimal ultrasonic radiation conditions for a particular material can maximize particle dispersion; thus, it is crucial to determine the ultrasonic irradiation energy required to generate a stable state using ultrasonic dispersion. The stability of TiO_2_ colloids was investigated for different DSE values. The occurrence of particle re-agglomeration was determined by analyzing the particle size distribution over time. Figure 10 shows the particle size distribution of the TiO_2_ colloids for various DSE values, where each DSE value corresponds to a different exposure time. Figure 10a displays the particle size distribution of the TiO_2_ colloid at an ultrasonic exposure time of 15 min and DSE of 234 J/mL. The average particle size immediately after 10 min of ultrasonic irradiation was 0.181 μm. After seven days of ultrasonic dispersion, the particle sizes increased to between 0.2 and 1 μm. After 17 days, the average particle size was 0.296 μm, suggesting that the particle should be agglomerated. These results indicate that a dispersion time of 15 min with a DSE value of 234 J/mL is insufficient to maintain the dispersion stability of the TiO_2_ colloid (1 wt%). There was a significant number of sub-micron size particles in the particle size distribution immediately after dispersion, which suggests that 234 J/mL is not the DSEcr value of the TiO_2_ colloid (1 wt%).

Figure 10b shows the particle size distribution of the TiO_2_ colloid with an ultrasonic exposure time of 84 min and a DSE value of 1363 J/mL. Table 2 shows the average particle size for each sample. The average particle size immediately after 84 min of ultrasonic irradiation was 0.07 μm, which increased to 0.2−1 μm after seven days; further, an average particle size of 0.092 μm was observed, which basically implied an increase of 0.02 μm. After 17 days, the average particle size was 0.186 μm, suggesting that more agglomerated particles were distributed in the sample. We may conclude that a DSE value of 1363 J/mL, corresponding to 84 min of ultrasound exposure, was also insufficient to maintain the dispersion stability of the TiO_2_ colloid.

Figure 10c shows the particle size distribution of the TiO_2_ colloid with an ultrasonic exposure time of 120 min and a DSE value of 1947 J/mL. After irradiating the dispersion of the TiO_2_ colloid with an ultrasonic wave energy of 1947 J/mL, the stability of the sample was assessed for up to 30 days to check if the stability lasted longer. The distribution was uniform in this case, with an average particle size of 0.068 μm, showing that agglomeration does not occur over time. It could be concluded that the TiO_2_ colloid (1 wt%) maintains dispersion stability under these conditions. Therefore, the DSEcr of the TiO_2_ colloid (mean particle size = 0.068 μm) can be concluded to be 1947 J/mL.

Thus, ultrasonic dispersion, triggered by the energy generated during the collapse of cavitation, disintegrated the agglomerated particles in the colloid and distributed them into small and uniform particles. As a result, the Brownian motion was faster than the settling velocity of the particles, which limited the time period of precipitation and re-agglomeration and facilitated maintaining the dispersion stability.

## 5. Conclusions

This study used a focused ultrasound technique to investigate a method for manufacturing surfactant-free waterborne TiO_2_ nanoparticles. The optimal conditions for preparing the surfactant-free waterborne 1 wt% TiO_2_ nanoparticles at the 100 nm scale with a dispersion stability of 30 days were determined to be 396 kHz and 102 W with the DSEcr value of 1947 J/mL. Because the focused ultrasound technique uses a strong sound field focused at the center, it is highly efficient in dispersing nanoparticles; this results in a well-dispersed and narrow particle size distribution of the waterborne nanoparticles with a long-term dispersion stability and an average particle size of ≤100 nm, in contrast with conventional ultrasound dispersion methods. After focused ultrasound dispersion, a stable zeta potential was obtained, and a stable colloidal state was maintained without any re-aggregation of nanoparticles in the colloid. The TiO_2_ colloids dispersed under these conditions can be applied to various products, such as cosmetics, paints, inks, and foods. Furthermore, as the TiO_2_ colloids are dispersed as nanoparticles under surfactant-free conditions, they can be used in high-performance products. Because the results are highly affected by the temperature and handling of the ultrasound equipment, subsequent studies will be conducted to further optimize and standardize the equipment.

## Figures and Tables

**Table 1:** Physical properties of the nanoparticles, solvent, and suspension used in the experiment.

Nanoparticle	Solvent	Suspension
TiO_2_ (P25) Degussa (Evonik)	Deionized Water	TiO_2_ colloid
Mean diameter: 25 nm	Resistivity: 18.2 MΩ·cm	Concentration: 1 wt%
Density: 3.78 g/cm^3^	pH: 7.2–7.6	Volume: 100 mL
		Pre-treatment: none
		pH: 4.9–5.2

**Table 2:** Delivery of sonic energy (DSE), average particle size, and polydispersity index (PDI) for different ultrasonic exposure times (f = 396 kHz and P_set_ = 102 W).

Exposure Time (min)	DSE [J/mL]	Mean (μm)/PDI		
		After 10 Min	After 7 Days	After 17 Days
15	234	0.181/2.684	0.213/3.125	0.296/4.931
42	682	0.092/1.996	0.101/2.132	0.142/2.384
84	1363	0.070/1.663	0.092/1.862	0.186/2.464
99	1616	0.071/1.654	0.073/1.712	0.090/1.990
108	1753	0.067/1.526	0.069/1.519	0.091/1.993
120	1947	0.068/1.518	0.067/1.516	0.068/1.515

**Table 3:** Changes in DSE, pH, and zeta potential with increasing ultrasonic exposure time.

	Sample 1	Sample 2	Sample 3	Sample 4
Ultrasonic exposure time (min)	0	15	84	120
DSE (J/mL)	0	234	1363	1947
pH	5.1	4.7	4.2	3.8
Zeta potential (mV)	19.6	29.2	38.6	43.1

## Data Availability

Not applicable.

## Supplementary Materials

The following are available online at https://www.mdpi.com/2079-4991/11/2/427/s1, Figure S1: X-ray diffraction pattern (a) before and (b) after ultrasonic dispersion.
