# Supplementary material for: Novel Surfactant-Free Water Dispersion Technique of TiO2 NPs Using Focused Ultrasound System
Source: Nanomaterials (Basel). 2021 Feb 8;11(2):427. doi: 10.3390/nano11020427 (PMC7915381; doi:10.3390/nano11020427)
Supplement: Supplementary file 1 [file nanomaterials-11-00427-s001.pdf]

nanomaterials

Supplementary Material

Novel Surfactant-Free Water Dispersion Technique of TiO_2 NPs Using Focused Ultrasound System

Seon Ae Hwangbo, Minjeong Kwak, Jaeseok Kim and Tae Geol Lee *

Nanosafety Team, Safety Measurement Institute, Korea Research Institute of Standards and Science (KRISS), 267 Gajeong-ro, Yuseong-gu, Daejeon 34113, Korea; hbsa@kriss.re.kr (S.A.H.); kwakmj@kriss.re.kr (M.K.); jaeseok.kim@kriss.re.kr (J.K.)

* Correspondence: tglee@kriss.re.kr; Tel.: +82-42-868-5003

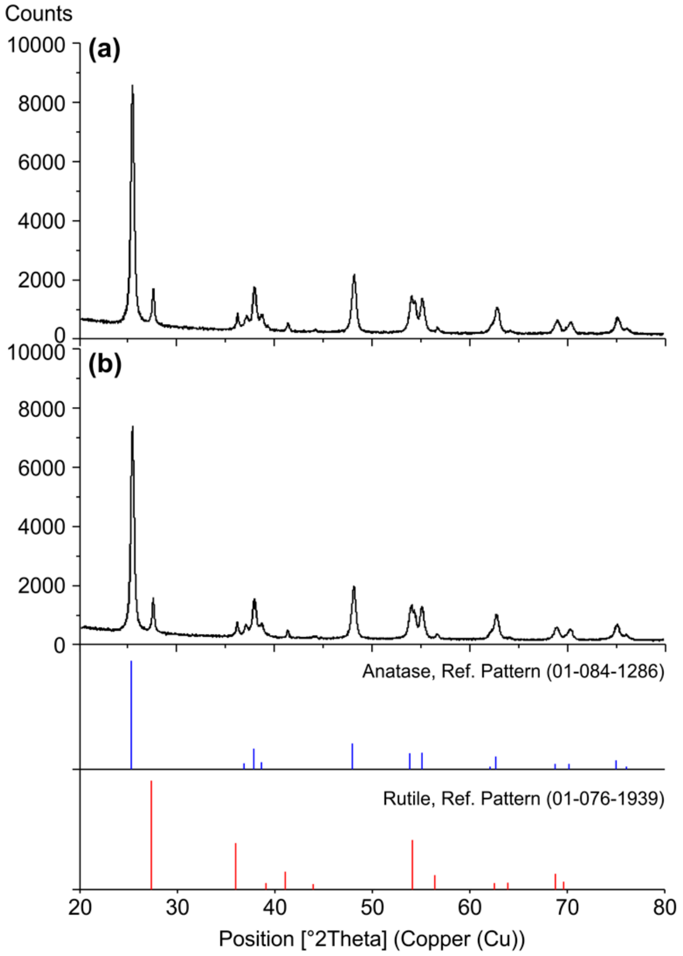

Figure S1. X-ray diffraction pattern (**a**) before and (**b**) after ultrasonic dispersion.
